# Bilateral Gluteus Maximus Tendon Tear Following an Apparently Low-Impact Trauma: A Case Report

**DOI:** 10.7759/cureus.81490

**Published:** 2025-03-31

**Authors:** Amrit Samra, Ayley K Loh, Alastair Littlewood, Samson O Oyibo

**Affiliations:** 1 Acute Medicine, Peterborough City Hospital, Peterborough, GBR; 2 Medicine, Peterborough City Hospital, Peterborough, GBR; 3 Radiology, Peterborough City Hospital, Peterborough, GBR; 4 Diabetes and Endocrinology, Peterborough City Hospital, Peterborough, GBR

**Keywords:** elderly trauma, gluteus maximus muscle, hip joint pain, loin pain, low-impact trauma, pelvic mri, tendon tear

## Abstract

Gluteus maximus tendon tear is an uncommon condition, probably due to the stout nature of its tendon structure as well as the potential for this type of injury to be overlooked by the attending healthcare worker. We present a case of bilateral gluteus maximus tendon tear in an 89-year-old female patient who presented with severe right buttock pain radiating to her right hip, groin, and thigh that started after sitting heavily on a stool. The bilateral tendon tear was discovered when she had a magnetic resonance imaging scan performed to rule out any occult hip or pelvic fracture that may have been missed during a previously performed X-ray and computed tomography scan. This case illustrates the importance of thorough history taking and examination and having a heightened sense of awareness when it comes to gluteus maximus tendon tear in a patient presenting with persistent traumatic pelvic or hip pain despite no fracture demonstrated on initial imaging.

## Introduction

The gluteus maximus is the most superficial muscle of the posterior pelvic girdle. It contributes to the contour of the buttocks and serves as the primary extensor of the hip joint. It is essential for maintaining an erect posture and facilitating movements, such as rising from a seated position and climbing stairs [1]. Anatomically, the gluteus maximus originates at the latero-posterior surface of the sacrum and coccyx, the gluteal surface of the ilium, the thoracolumbar fascia, and the sacro-tuberous ligament. Distally, approximately two-thirds of its proximal fibers insert into the iliotibial band, with the remaining fibers attaching to the gluteal tuberosity of the femur bone. It is innervated by the inferior gluteal nerve and gets its blood supply from the inferior and superior gluteal arteries. Its main functions are extension, external rotation, abduction, and adduction of the thigh [1]. Deep to the gluteus maximus muscle are two smaller gluteus muscles (gluteus medius and gluteus minimus), which both abduct and internally rotate the thigh.

Isolated injuries to the gluteus maximus muscle or tendon are rare. They are often identified incidentally during imaging for other gluteal pathologies or during orthopedic surgery, for example, during total hip joint replacement. This could be due to the stout nature of the tendon structure as well as the potential for this injury to be overlooked by the attending doctor, especially when the tendon tear is small or symptoms are vague [2]. Injury to the gluteus maximus can result from direct trauma, excessive force, or sudden movements leading to muscle strain or tear. Such injuries may cause significant functional impairment, including pain, swelling, and reduced range of motion. Despite the size and functional importance of the gluteus maximus, documented cases of isolated gluteus maximus tendon tear are exceedingly uncommon compared to injuries to the gluteus medius and gluteus minimus, where injuries are thought to be due to the degenerative process of the trochanteric enthesis (muscle-tendon-bone unit) rather than a primary traumatic tear [3-5]. Isolated gluteus maximus injury from trauma or impingement have been reported in athletes performing high-demand activities [6].

To the best of our knowledge, isolated gluteus maximus tendon tear has rarely been reported; there are only a handful of cases in the literature [7-10]. There are no previous reports of bilateral gluteus maximus tendon tear. We describe a case that illustrates these points. This case involves an 89-year-old female who sustained a bilateral gluteus maximus tendon tear secondary to an apparently low-impact trauma. This case underscores the need for heightened clinical awareness and prompt diagnosis of gluteus maximus tendon tear, particularly in the elderly population, to ensure appropriate management and optimal functional recovery. Written consent was obtained from the patient before submitting this case report.

## Case presentation

Medical history and demographics

An 89-year-old female patient presented to the Emergency Department with severe right buttock pain, which started after sitting heavily on a stool whilst preparing for bed the night before. The pain radiated to her right hip, groin, and down to her right thigh. The pain was immediate and sharp, rendering her unable to rise from the stool or mobilize effectively. She could shuffle backward with difficulty, but she was unable to shuffle forward or sideways. She managed to use a stairlift to relocate downstairs and spent the night in a chair. There were no other associated symptoms, such as headache, fever, cough, or diarrhea. Her past medical history included gastroesophageal reflux disease, hypertension, chronic kidney disease, chronic mild anemia, a previous deep vein thrombosis in the right leg, pulmonary embolism, gallbladder disease, osteoarthritis, osteoporosis, polymyalgia rheumatica, hypothyroidism, abdominal aortic aneurysm, and recurrent urinary tract infections. Her regular medications included alendronic acid, amlodipine, levothyroxine, nitrofurantoin, omeprazole, and rivaroxaban. She was sensitive to trimethoprim, amitriptyline hydrochloride, etoricoxib, and cimetidine. She lived independently, mobilized unaided indoors but used a mobility scooter outdoors.

On examination, the patient was alert and comfortable, with a temperature of 36.5°C, a heart rate of 80 beats per minute, respiratory rate of 16 breaths per minute, a blood pressure of 139/54 mmHg, and oxygen saturation of 96% on room air. The right hip was tender on palpation, but no bruising was observed over the area. There were no signs of bruising over her back, buttocks, or thighs. She was only able to straight-leg raise her right leg to twenty degrees off the bed before experiencing severe pain. The left leg had no abnormal findings. No weakness was demonstrated in her lower limb muscles. All pulses were palpable. She weighed 91.9 kg with a body mass index of 35.0 kg/m2.

Investigations

The initial investigations revealed a slightly reduced hemoglobin level consistent with her chronic mild anemia and a coagulation profile consistent with the use of anticoagulant therapy. The markers of muscle injury, namely, serum lactate dehydrogenase, was slightly elevated, and creatinine kinase was normal (Table 1).

**Table 1:** Initial blood test results.

Blood parameters	Results	Reference range
Hemoglobin (g/L)	107	115-165
White cell count (10^9^/L)	9.1	4-11
Platelet count (10^9^/L)	293	150-400
Sodium (mmol/L)	139	133-146
Potassium (mmol/L)	4.2	3.5-5.3
Chloride (mmol/L)	105	95-108
Creatinine (µmol/L)	80	59-104
Urea (mmol/L)	6.8	2.5-7.8
Total bilirubin (µmol/L)	4	<21
Alkaline phosphatase (U/L)	65	30-130
Alanine transferase (U/L)	16	10-60
Adjusted calcium (mmol/L)	2.47	2.2-2.6
Thyroid stimulating hormone (mU/L)	1.26	0.3-4.2
Lactate dehydrogenase (U/L)	258	<250
Creatinine kinase (IU/L)	85	25-200
Prothrombin time (seconds)	22.4	9.4-16.4
Activated plasma thromboplastin time (seconds)	36.5	24-26

**Figure 1:**
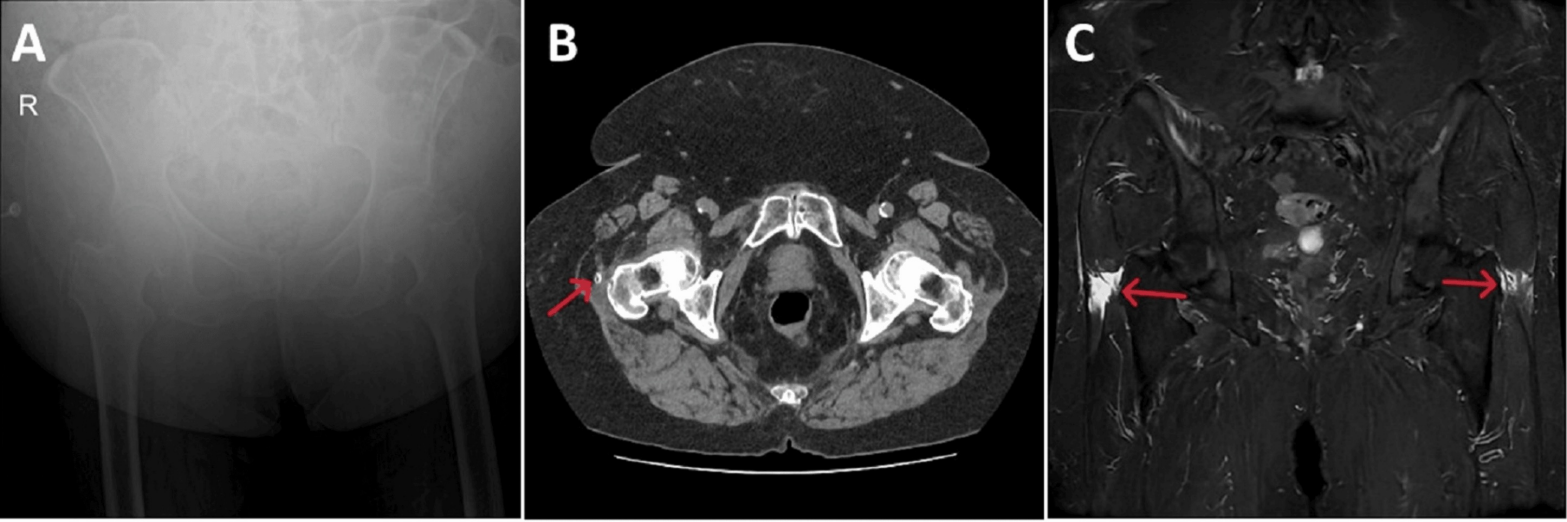
Radiological images of the pelvic, hip joint, and femur bones. A: Suboptimal X-ray film showing no fractures. B: Computed tomography scan of the pelvis at the level of the greater trochanter (transverse view) demonstrating enthesopathy near the right greater trochanter and some calcifications associated with the gluteus medius tendon (red arrow). C: Magnetic resonance scan of the pelvis at the level of the greater trochanter (coronal view) demonstrating bilateral gluteus maximus tendon tear with evidence of edema indicating that these were acute events (red arrows).

An initial X-ray of the pelvic and femur bones was suboptimal for bony detail but did not reveal any fractures. A computed tomography (CT) scan of the pelvis also ruled out acute pelvic or hip fractures but noted some enthesopathy near the right greater trochanter. Because of the continued pain, a magnetic resonance imaging (MRI) scan of the pelvis was performed to rule out an occult fracture. This demonstrated bilateral gluteus maximus tendon tear with evidence of edema, indicating that these were acute events; worse on the right-hand side (Figure 1).

Treatment

Paracetamol 1000 mg four times a day and codeine phosphate 60 mg four times a day were given for suspected traumatic right hip bone injury. Low-dose liquid morphine was available if the patient needed it. Because of a history of polymyalgia rheumatica, she was initially started on prednisolone 5 mg daily in case this was an exacerbation. The prednisolone was stopped once the MRI scan confirmed the diagnosis of a traumatic bilateral gluteus maximus tendon tear.

Outcome and follow-up

While on adequate analgesia, the patient used a walker-frame for mobilizing. After 10 days of physiotherapy, weight-bearing, and physical aids, she improved and was able to mobilize effectively without assistance. She had a telephone consultation a week later and reported that she had a complete resolution of symptoms and had regained independence in activities of daily living (not requiring any aids). Therefore, the total time from injury to resolution of symptoms was three weeks.

## Discussion

We have reported an elderly female patient who sustained a traumatic bilateral gluteus maximus tendon tear after minor trauma. She had severe buttock pain on the right-hand side but no symptoms on the left-hand side, consistent with the larger tear on the right-hand side. Additionally, there were no visible signs of bruising. The bilateral gluteus maximus tendon tear was discovered during imaging to rule out an occult hip or pelvic fracture following normal X-ray and CT scan imaging amidst continuous unexplained trauma-induced pelvic pain. We noted the slightly elevated lactate dehydrogenase level with a normal creatinine kinase level. This was likely due to the fact that there was a tendon tear rather than a muscle injury 

There are just a handful of case reports of patients who have sustained a gluteus maximus muscle tendon tear [7-10]. These cases are illustrated in Table 2.

**Table 2:** Reported cases of gluteus maximus muscle tendon tear.

Author's name, publication year	Patient demographics	Presentation	Treatment	Outcome
Kara et al., 2015 [7]	52-year-old male	Severe pain in right buttock radiating down leg after jumping from a lorry	Rest, ice-pack, and a non-steroidal anti-inflammatory drug	Full recovery within a few days
Shekhbihi et al., 2022 [8]	63-year-old male	Stabbing pain in the right proximal thigh during water aerobics training	Open surgical repair	Full recovery at three months
Alradwan et al., 2022 [9]	36-year-old male	Posterior thigh pain after a fall	Open surgical repair	Full recovery at three months
King et al., 2024 [10]	72-year-old male	Left buttock pain after a fall while playing pickleball	Physical therapy, ice-pack, and a non-steroidal anti-inflammatory drug	99% recovery in 4 weeks

The above handful of case reports demonstrates that gluteus maximus tears can result from several different types of traumas, they can affect any age, and affected patients can have variable presentations such that a high index of suspicion is required to make a diagnosis. All the cases had a history of some type of trauma and were diagnosed by MRI scan after having had a normal-appearing X-ray. Of note, the patients in two of the four case reports required open surgical repair [8,9]. Our patient's demographics included being elderly and less physically active. Additionally, the bilateral tear was deemed not severe enough to warrant surgery, and she fully recovered (symptomatically and physically) with conservative management involving pain control and physiotherapy.

This is the first report of a patient sustaining a bilateral gluteus maximus tendon tear after minor trauma. Possible risk factors in this patient’s case could include age-related muscular tendon degeneration and being overweight: the trauma was perceived as low-impact but could really be high-impact because of her body weight. The CT scan also indicated enthesopathy, which could be related to inflammation, sarcopenia, and calcification, which could all contribute to tendon weakness in the elderly. Another interesting feature in this case was the presence of an asymptomatic tendon tear on the left side. This finding of an asymptomatic tear brings into question the true incidence of gluteus maximus tendon tear.

There are no significant outcome studies in the literature, given the rarity of an isolated gluteus maximus tendon tear. One manuscript that discussed the surgical repair of gluteus maximus tendon tears emphasized the importance of thorough history taking, examination, and imaging in making a diagnosis [11]. MRI is the imaging method of choice when investigating and managing musculoskeletal pathologies [12]. The initial management should be conservative, involving an extensive course of physical therapy or a home-exercise program, oral anti-inflammatory drugs, injections (corticosteroids or platelet-rich plasma), and shockwave therapy [11,13]. Open surgical repair should only be reserved for patients who, despite extensive non-operative management, still present with recalcitrant symptoms or for patients with full-thickness tears [11,14].

## Conclusions

This is the first report of a case of a bilateral isolated gluteus maximus tendon tear occurring after minor trauma. Isolated gluteus maximus tendon tear is rare and should be considered as a differential diagnosis when assessing painful hip and buttocks conditions, especially when symptoms persist after a traumatic injury and the X-ray does not detect any pelvic or hip bone fracture. We recommend considering MRI in elderly patients with unexplained buttock or hip pain and normal radiographs, especially if functional impairment is disproportionate to clinical findings. The importance of thorough history taking and examination cannot be overstated. The management of gluteus maximus tendon tear is largely conservative; open surgery is reserved for recalcitrant cases and full-thickness tears. We hope that this case report not only contributes to the literature but also raises awareness of traumatic gluteus maximus tendon tear.
